# Physiological Characterisation of Severe Heart Failure Using Long-Duration Ambulatory ECG: A Retrospective Exploratory Analysis

**DOI:** 10.7759/cureus.102943

**Published:** 2026-02-04

**Authors:** Junaid Aamir Khan, Usman Ali, Md Tanzim Ahsan, Ratan Chandra Roy, Opeyemi S Alamu, Francesco Alessi Longa

**Affiliations:** 1 Department of Acute Medicine, Wrightington, Wigan and Leigh Teaching Hospital, NHS Foundation Trust, Wrightington, GBR; 2 Department of Elderly Medicine, University Hospitals Sussex, Worthing, GBR; 3 Department of Physical Medicine and Rehabilitation, Dhaka Medical College Hospital, Dhaka, BGD; 4 Department of Statistics, Federal College of Animal Health and Production Technology Ibadan, Ibadan, NGA; 5 Department of International Law, Azteca University, Chalco, MEX

**Keywords:** ambulatory ecg, digital health, heart failure, heart rate variability, remote monitoring, wearable devices

## Abstract

Background: Heart failure is associated with progressive autonomic and cardiorespiratory dysregulation that is incompletely captured by episodic clinical assessment. Continuous ambulatory ECG offers a unique opportunity to describe longitudinal physiological dynamics, particularly in patients with advanced disease.

Aim: The aim of this study is to provide a descriptive and exploratory physiological characterisation of cardiac and respiratory dynamics in patients with severe heart failure using long-duration ambulatory ECG recordings.

Methods: This retrospective secondary analysis used the publicly available Beth Israel Deaconess Medical Center (BIDMC) Congestive Heart Failure database, comprising approximately 20 hours of two-channel ambulatory ECG recordings from 15 patients with NYHA class III-IV heart failure. Automated ECG annotations were used to derive heart rate (HR), time- and frequency-domain heart rate variability (HRV), and ECG-derived respiration (EDR). Analyses focused on inter-individual variability and within-subject temporal patterns using descriptive statistics and longitudinal visualisation.

Results: Marked heterogeneity was observed across subjects in mean HR and HRV metrics, with generally depressed HRV consistent with advanced autonomic dysfunction. Longitudinal analyses revealed substantial within-subject variability and temporal trends in HR and EDR across recording periods, including systematic differences between early and late segments. HR and HRV showed partial dissociation across individuals, underscoring the limitations of single-parameter assessment.

Conclusion: Long-duration ambulatory ECG enables rich descriptive characterisation of cardiac and respiratory physiology in severe heart failure. These findings are exploratory and hypothesis-generating, providing a physiological foundation for future studies that integrate clinical covariates, outcomes, and contemporary wearable technologies.

## Introduction

Heart failure (HF) is a long-term and progressive clinical condition of the heart, which occurs due to the inability of the heart to supply the body with its needs in terms of metabolism. It is also a significant health burden to the world, causing more than 64 million cases every year, with a rising number related to the ageing of the population and better survival following acute cardiovascular incidents [1]. Despite the improvements in the guideline-based medical treatment and medical device-based interventions, HF is still linked to high morbidity, mortality and a large number of readmissions to the hospital. The readmission rate of patients hospitalised with HF is about 1 in 5 within 30 days, showing ongoing difficulties with the management of the disease over the long term and post-discharge monitoring [2,3].

Despite growing interest in wearable and remote monitoring technologies for HF, there remains a lack of detailed descriptive analyses of long-duration physiological dynamics in patients with advanced disease using open-access data. Most prior studies focus on prediction, intervention, or device validation rather than foundational physiological characterisation. This limits understanding of inter-individual heterogeneity and within-subject temporal variability that underpin remote monitoring strategies. Subtle changes in the cardiovascular and autonomic functioning, which over time may occur in days or weeks, are pre-conditional to clinical decompensation. Yet, traditional models of monitoring are based mainly on intermittent outpatient visits, self-reports on symptoms, and occasional evaluation of vital signs, which cannot give many clues about the dynamic character of HF development [4]. This leads to aspects of preventable hospitalisations and poor outcomes, and often goes unnoticed as an early warning sign of worsening HF [5].

It has been suggested to use remote monitoring technologies to overcome these limitations. Remote monitoring provides the ability for continuous or almost continuous measure of physiological parameters in the non-clinical setting, helping clinicians to monitor patient status over time as opposed to individual measurements [6]. On implantable as well as non-invasive monitoring systems, between five and six studies have been conducted on HF populations, with a number showing that the type of HF-related hospitalisation reduced when data on remote monitoring were effectively incorporated into clinical care processes [7,8]. Recently, wearable and ambulatory biosensors have become the focus of attention with the aim of monitoring over the long term, in scalable and less invasive devices.

Ambulatory and wearable biosensors have the potential to record physiological signals that are strongly associated with HF pathophysiology, such as heart rate (HR), heart rate variability (HRV), respiratory variations, and circadian rhythm parameters. Vascular changes in the functioning of the autonomic nervous system, which are manifested by a decrease in HRV and the loss of circadian regulation, are also clearly described characteristics of advanced HF and have been linked to the severity and prognosis of the disease [9-11]. Repeated evaluation of such parameters provides the possibility of recording physiological instability at its initial stage before the development of apparent symptoms.

One of the oldest and most established forms of continuous remote physiological surveillance is long-term ambulatory electrocardiogram (ECG) monitoring. An ambulatory ECG is used to record cardiac electrical activity at very high resolution over prolonged durations, providing the ability to analyze HR dynamics, HRV, and ECG-derived respiration. Most of the physiological indicators provided by wearable devices to date are based on the same principles as had been initially discovered in the ambulatory ECG study, which highlights the continued applicability of such data to modern contexts of remote monitoring [12,13].

There is limited access to long-duration and high-quality physiological data of patients with severe HF, especially in open and reproducible studies. The Beth Israel Deaconess Medical Center (BIDMC) Congestive Heart Failure Database is an ambulatory ECG long-term recording of nursing data on patients with New York Heart Association (NYHA) class III-IV HF hosted on PhysioNet that offers a single chance to investigate the dynamics of cardiac and autonomic functioning in a group that is at risk [14]. Accordingly, this study is not designed to assess clinical outcomes, device performance, or intervention efficacy. Instead, it provides a retrospective exploratory characterization of cardiac and respiratory physiological signals derived from continuous ECG, intended to inform hypothesis generation and guide future outcome-linked or wearable-based investigations. The use of PhysioNet resources has been shown to be useful in the past in researching HRV, nonlinear dynamics of the cardiovascular system, and cardiorespiratory interaction in cardiovascular disease [15].

We conduct a secondary analysis of the BIDMC Congestive Heart Failure Database in this paper to model the continuous physiological measures applicable to wearable-based remote sensors. The primary aim of this study is to descriptively characterize inter-individual variability and within-subject temporal dynamics of HR, HRV, and ECG-derived respiration in patients with severe HF.

## Materials and methods

Study design and data source

This paper was developed in the form of a secondary retrospective study of publicly available physiological data retrieved via the BIDMC Congestive Heart Failure Database available in PhysioNet. The database holds long-term ambulatory electrocardiogram (ECG) of patients with severe congestive heart failure (CHF). All information will be entirely de-identified and will be open-access licensed; thus, this analysis did not require ethical approval or informed consent.

Study population

The data consist of recordings of 15 patients diagnosed with severe CHF who were classified as NYHA functional classes III and IV. Important clinical variables, including cardiac rhythm, medication use, and comorbidities, were unavailable in the dataset and represent major potential confounders, particularly for HRV interpretation.

The group includes 11 male patients aged 22-71 years and four female patients aged 54-63 years. All the recorded data were collected before the oral administration of milrinone became effective in conducting a larger clinical study. This segment is a sample of patients with severe symptomatic HF, a group of people that is at a high risk of physiological instability and poor consequences. An ambulatory monitoring system refers to a computer-based system that allows physicians to monitor patients remotely without the need to be physically present with them.

Inclusion Criteria

Participants were included if they were diagnosed with severe CHF classified as NYHA functional class III or IV, had long-duration ambulatory electrocardiogram (ECG) recordings available in the BIDMC CHF Database, and had recordings of sufficient signal quality to allow continuous heart rate and heart rate variability analyses.

Exclusion Criteria

Participants were excluded if ECG recordings were incomplete, excessively corrupted by artefacts, or lacked automated annotation files required for RR interval analysis. No additional clinical exclusions were applied, as this was a secondary retrospective analysis of an existing de-identified dataset.

Ambulatory monitoring system

An ambulatory monitoring system is a computer-based system that enables physicians to monitor patients remotely without necessarily being physically present with them. Each participant was monitored for a period of 20 hours to undergo continuous ambulatory ECG. The portable ambulatory ECG recorders recorded at Beth Israel Hospital were used to obtain recordings. Recording was done on two simultaneous ECG channels with a sample rate of 250 HZ and 12-bit resolution over a ±10 mV dynamic range. This was about 0.1-40 Hz in the decent recording bandwidth. The technical capabilities of these specifications are similar to and even higher than those of modern wearable ECG patches and biosensor-based monitoring devices.

Signal preprocessing

Raw ECG signals were handled with the standard signal processing techniques in physiology. R-peak detection was based on automated annotation files provided with the dataset and was not manually adjudicated. Automated ECG annotations may introduce errors in RR interval detection and derived HRV measures. Band-pass filtering was done to eliminate baseline wander and high-frequency noise. The R-wave peaks were automatically detected with the help of detection algorithms that were given along with the dataset annotations. Automatic generation of annotation files (that were not corrected manually) was also found in previous studies using this database. This was done to ensure that all the available signal sections were incorporated into the analysis to maintain the non-stop character of monitoring. The reliance on automated ECG annotations without manual verification may introduce systematic errors, particularly in HRV metrics sensitive to artefact and ectopy.

Feature extraction

The processed ECG signals were turned into physiological characteristics that may be relevant in remote HF monitoring.

Heart rate

The rate was calculated from the interval between one consecutive R and another R throughout the duration of the recording.

Heart rate variability

HRV indices were calculated as time-domain and frequency-domain to describe the regulation of the autonomic nervous system.

ECG-derived respiration

Respiratory dynamics were estimated based on ECG morphology and changes in R-R interval using some established ECG-based respiration measures.

These characteristics constitute the fundamental physiological indices that are generally aimed at by contemporary wearable and afloat surveillance gadgets to monitor HF. HRV and ECG-derived respiration were treated as indirect physiological descriptors rather than direct measurements, particularly given the ambulatory and pathological context.

Analytical approach

**Figure 1:**
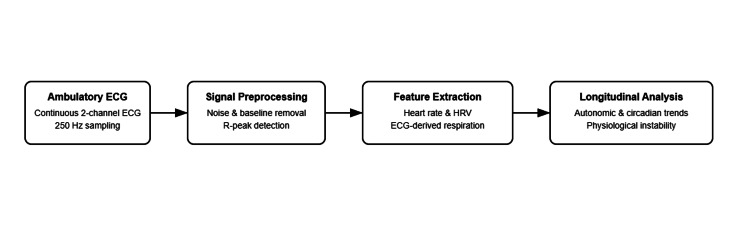
Remote physiological monitoring pipeline using ambulatory ECG data. This figure illustrates the analytical workflow used in this study. Continuous two-channel ambulatory ECG recordings from patients with severe HF were processed through a signal preprocessing stage, followed by feature extraction of HR, HRV, and EDR. These physiological metrics were then analysed longitudinally to characterise autonomic and cardiorespiratory dynamics relevant to wearable-based remote monitoring. HF: heart failure; HR: heart rate; HRV: heart rate variability; EDR: ECG-derived respiration.

An analysis was done in descriptive and exploratory ways to define inter-individual differences and changes through which physiological parameters vary over time. There was also continuous time-series data analysis on the identification of trends in autonomic dysfunction, circadian modulation, and cardiorespiratory coupling. Since there are no labelled clinical events (hospitalisation or mortality) in the data, physiological characterisation analyses and evaluations on feasibility, but not predictive modelling of clinical outcomes, were performed (Figure 1).

Statistical analysis

The exposures of physiological measures were summarized with descriptive statistics. The visual and quantitative evaluation of time-series trends was done during the period of monitoring of all subjects. A clinical endpoint test was not conducted because it was solely an exploratory study, and the sample size was too small. Standard scientific computing tools were used to carry out all of the analyses. All analyses were exploratory and descriptive in nature.

## Results

Data capture and analytics yield

Early-versus-late segmentation was used as a simple within-record longitudinal contrast to illustrate temporal variability rather than infer causality. The small cohort size (n = 15) limits generalizability, and findings should be interpreted as illustrative rather than representative [16,17]. Consistent with prior uses of this database, the annotation stream was treated as an automated R-peak series (not manually adjudicated), and results are interpreted as physiologic characterization rather than adjudicated clinical event detection [16,17].

Across subjects, the pipeline produced a per-subject RR interval series after physiologic plausibility filtering and robust outlier rejection, enabling calculation of time-domain HRV (mean NN, SDNN, RMSSD, and pNN50) and frequency-domain metrics (low frequency (LF), high frequency (HF), LF/HF) aligned to standard HRV reporting conventions [18]. Frequency-domain HRV was estimated from an interpolated tachogram using a Welch-style averaged periodogram approach [19,20].

Cohort-level descriptive statistics

Table 1 summarises cohort-level distributions of key physiologic features derived from the full recordings. Mean HR varied substantially across participants (range 63.00 to 123.73 bpm), reflecting marked inter-individual heterogeneity typical of severe CHF physiology in continuous ambulatory monitoring [16,19,21]. Time-domain HRV measures were generally low to moderate at the cohort level (median SDNN 0.06 s, median RMSSD 0.03 s), consistent with the well-described autonomic dysregulation in HF populations and with prior evidence linking lower HRV to worse prognosis in HF with reduced ejection fraction (HFrEF) [19].

**Table 1:** Descriptive statistics of derived physiological measures across subjects (n = 15). NN: normal-to-normal intervals; SDNN: standard deviation of normal-to-normal intervals; RMSSD: root mean square of successive differences; pNN50: percentage of successive NN intervals that differ by more than 50 ms.

Measure	Unit	Mean (SD)	Median [Q1, Q3]	Range
Mean HR	bpm	90.94 (15.79)	96.53 [77.58, 98.49]	63.00–123.73
SDNN	s	0.06 (0.03)	0.06 [0.05, 0.08]	0.02–0.10
RMSSD	s	0.04 (0.05)	0.03 [0.02, 0.04]	0.01–0.17
pNN50	%	9.49 (17.67)	2.69 [1.28, 5.55]	0.08–53.02
LF/HF	ratio	0.93 (0.76)	0.74 [0.57, 1.10]	0.23–3.46
EDR respiratory rate	breaths/min	8.90 (1.58)	8.79 [8.14, 9.02]	7.15–14.06

Interpretively, two patterns stood out. First, the cohort included a small subset of subjects with very high mean HR and markedly depressed HRV, exemplified by chf10 (mean HR = 124 bpm with very low SDNN), a physiological profile consistent with heightened sympathetic predominance or reduced vagal modulation, both frequently observed in advanced HF [22]. Second, a different subset showed higher short-term variability (higher RMSSD and pNN50), which can occur in ambulatory ECG depending on rhythm regularity, ectopy burden, and the balance of autonomic inputs across daily activity and sleep transitions [18,22].

Relationship between mean HR and HRV across subjects

**Figure 2:**
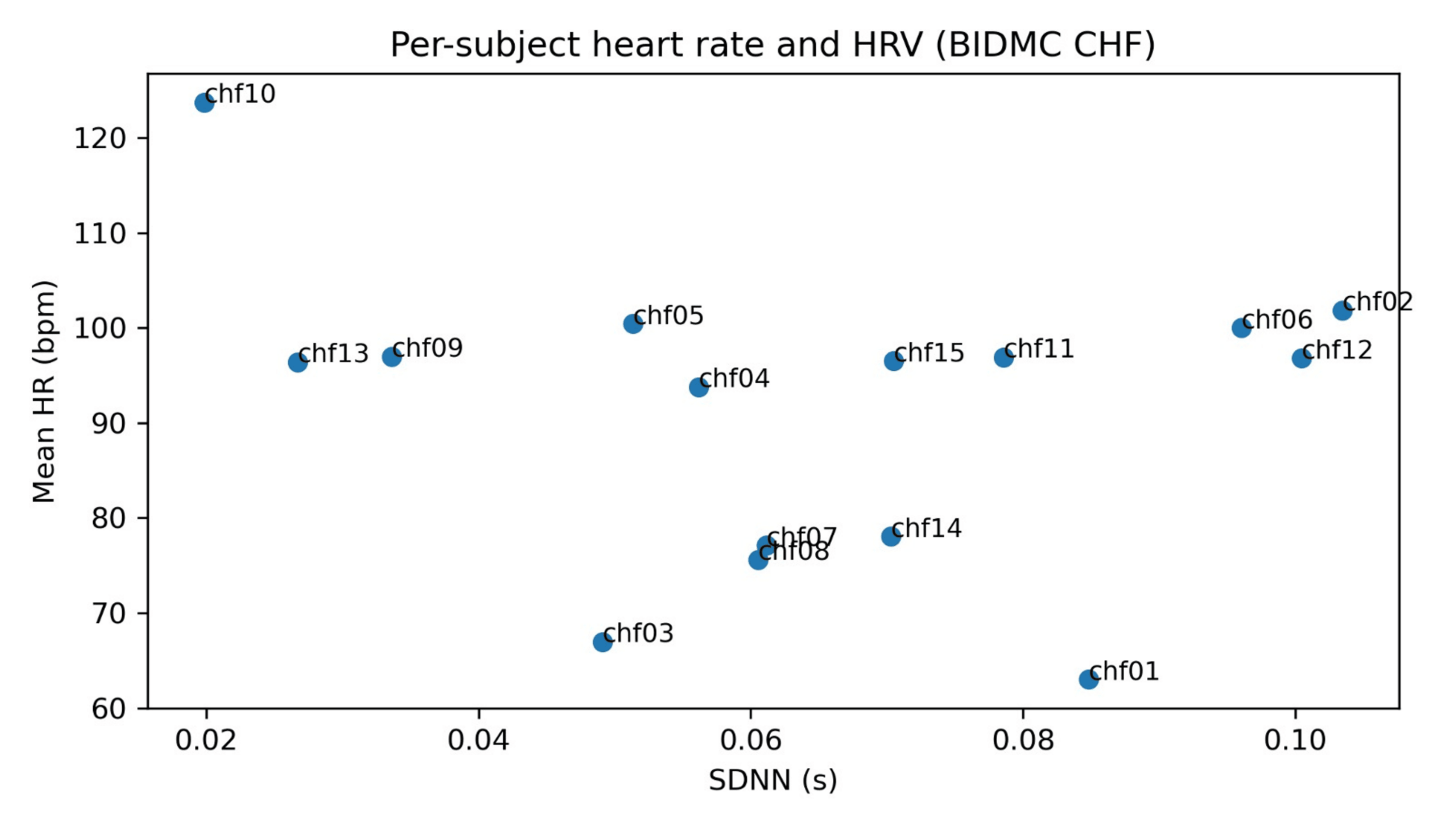
Per-subject heart rate and HRV (BIDMC CHF). BIDMC CHF: Beth Israel Deaconess Medical Center Congestive Heart Failure.

This dissociation is presented as a descriptive observation and should not be over-interpreted clinically in the absence of outcome data (Figure 2). In this cohort, mean HR and SDNN did not exhibit a strong linear alignment, underscoring that in severe CHF, HRV suppression can coexist with either tachycardia or more modest resting rates depending on medications, conduction disease, sleep-wake composition, and rhythm stability [21,23,24]. This observation supports a practical point relevant to wearable monitoring: single-parameter tracking (HR only) can miss clinically meaningful autonomic variability patterns, whereas multi-feature profiling (HR plus HRV dynamics) better reflects physiologic state [25,26].

Early-versus-late recording comparison

To operationalise a simple within-record longitudinal contrast, recordings were segmented into early and late thirds (relative time, because clock-time anchors are not supplied in the dataset). Across subjects, mean HR was higher early (mean 94.50 bpm) and lower late (mean 87.32 bpm), with an average change of −7.18 bpm (SD 7.63). Thirteen of 15 subjects showed a decrease from early to late segments, while two subjects showed modest increases. This consistent directional shift is compatible with expected ambulatory patterns where rest or sleep periods later in the recording contribute to lower average HR, while also illustrating that long-duration recordings contain meaningful within-person dynamics even without labelled outcomes [18,22,25].

**Figure 3:**
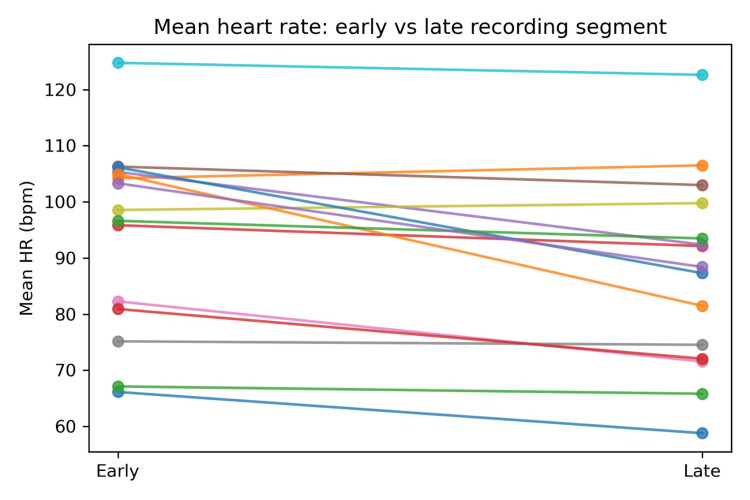
Mean heart rate: early versus late recording segment.

Figure 3 visualises these paired early-late differences per subject, emphasising that the magnitude of change varied widely (minimum = −23.45 bpm; maximum = +2.31 bpm). For remote monitoring design, the key implication is that baseline and trend are both informative: a single spot HR value is less interpretable than an individual’s trajectory across hours, particularly in CHF, where circadian modulation, autonomic instability, and activity tolerance can reshape physiologic baselines [26-28]. 

Frequency-domain HRV distribution and interpretation

**Figure 4:**
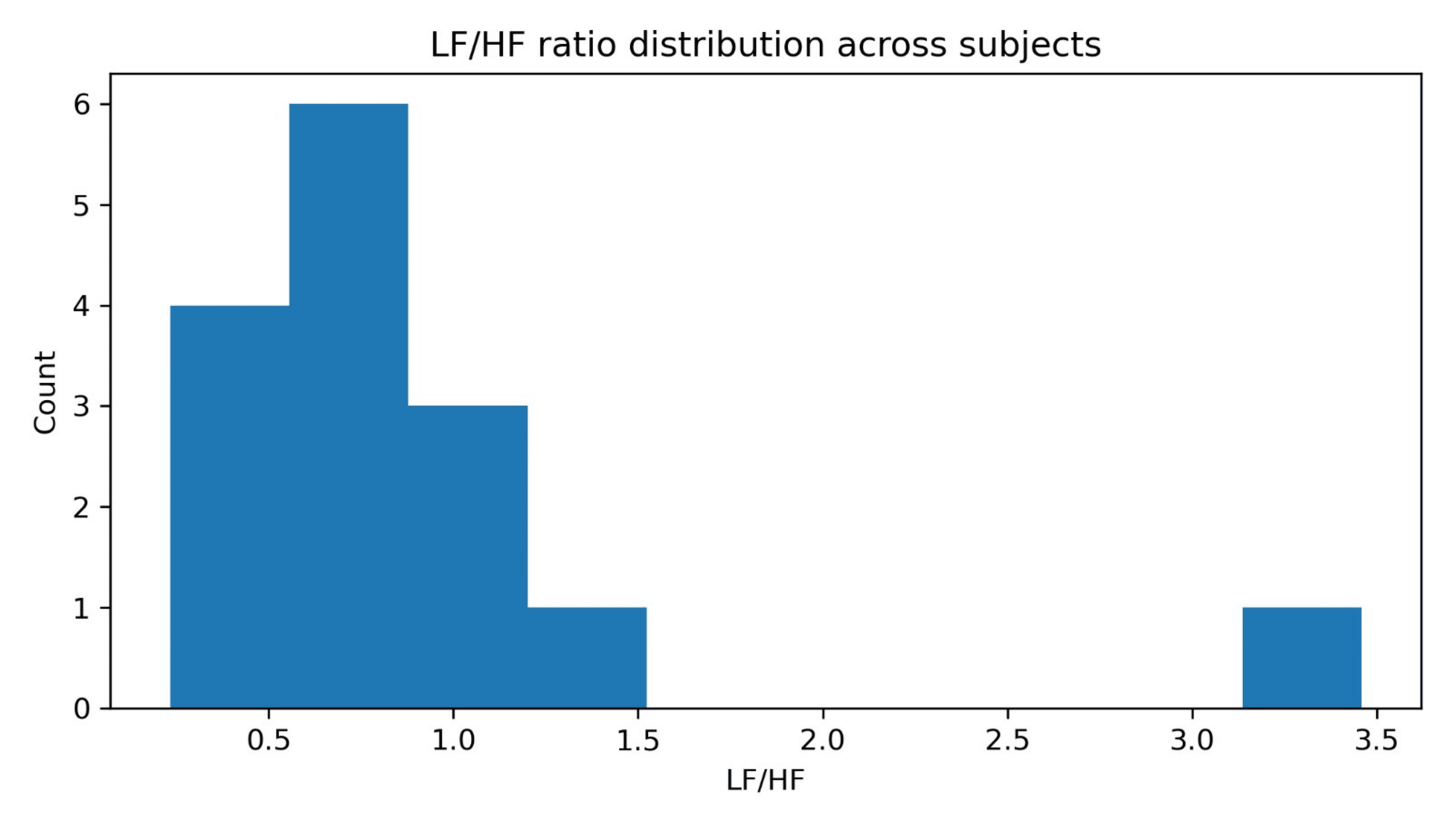
LF/HF ratio distribution across subjects. LF: low frequency; HF: high frequency.

LF/HF is reported as a spectral descriptor only and not as an indicator of sympathovagal balance, particularly in non-stationary and pathological ambulatory recordings. Across subjects, LF/HF ratios were centered below 1.0 (median 0.74, IQR 0.57 to 1.10), with one clear high outlier (maximum 3.46), producing the right-tailed distribution shown in Figure 4. In standard HRV reporting, LF and HF components are used as descriptive indices of oscillatory content in the RR series within predefined bands, but the physiological interpretation of LF/HF as "sympathovagal balance" is contested and should be treated cautiously, especially in disease states and in ambulatory conditions with non-stationarity [18,21]. Accordingly, LF/HF is presented here as a comparative spectral descriptor across subjects rather than a direct mechanistic biomarker. This positioning is consistent with contemporary recommendations that emphasize transparent reporting of preprocessing, stationarity considerations, and cautious inference, particularly when RR series include ectopy or rhythm irregularity [18,21,29].

Longitudinal five-minute window trends in HR and EDR

Windowed analysis (5-minute non-overlapping windows) was used to visualise within-subject temporal evolution of HR and an ECG-derived respiration (EDR) proxy. Across subjects, the typical within-subject dispersion over the recording was substantial: median windowed HR interquartile range was ~9.20 bpm, and the median windowed HR range was ~28.63 bpm, indicating broad fluctuations consistent with changes in activity and rest across ambulatory conditions [16,18].

**Figure 5:**
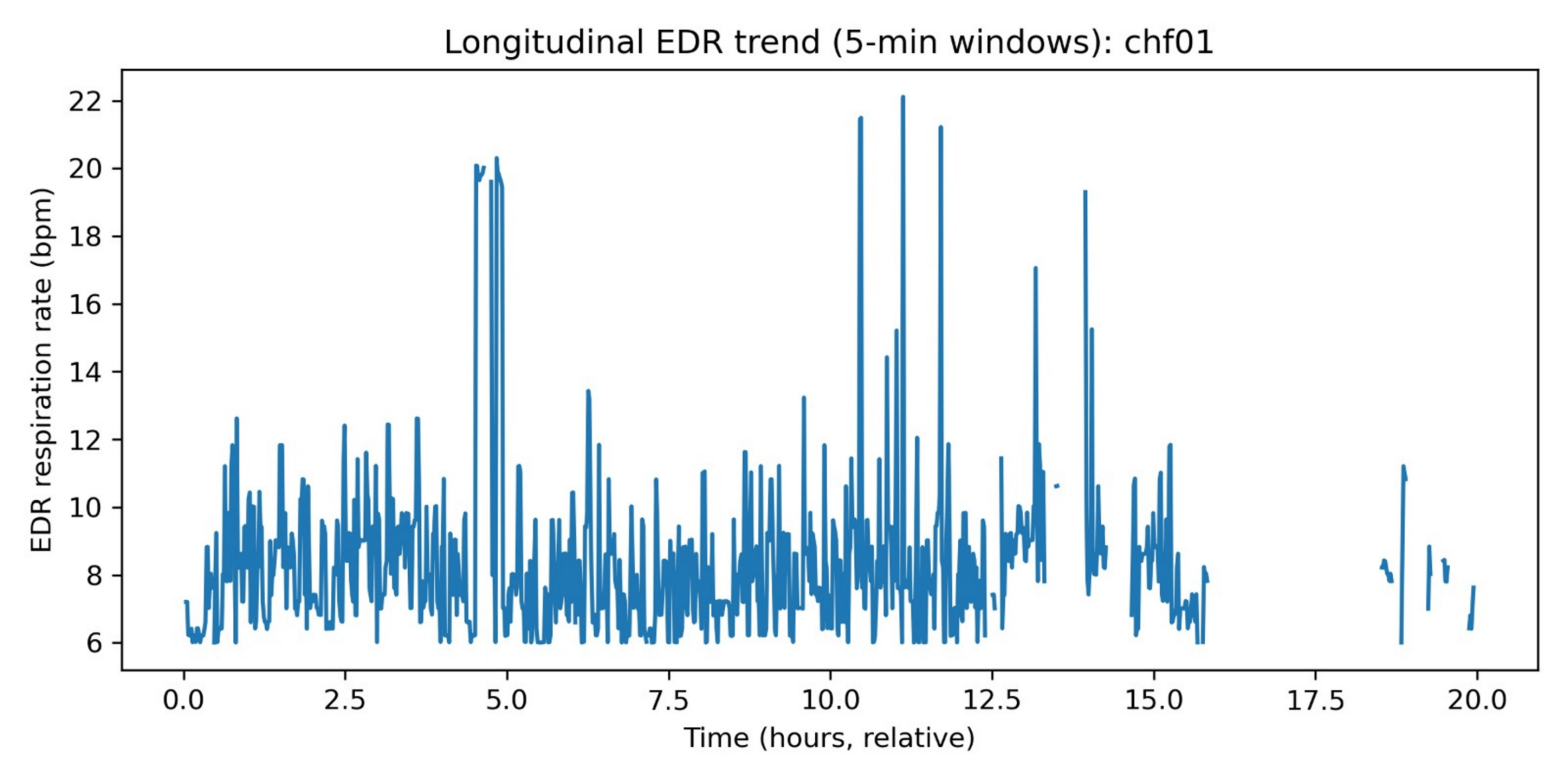
Longitudinal HR trend (five-minute windows). HR: heart rate; EDR: ECG-derived respiration.

**Figure 6:**
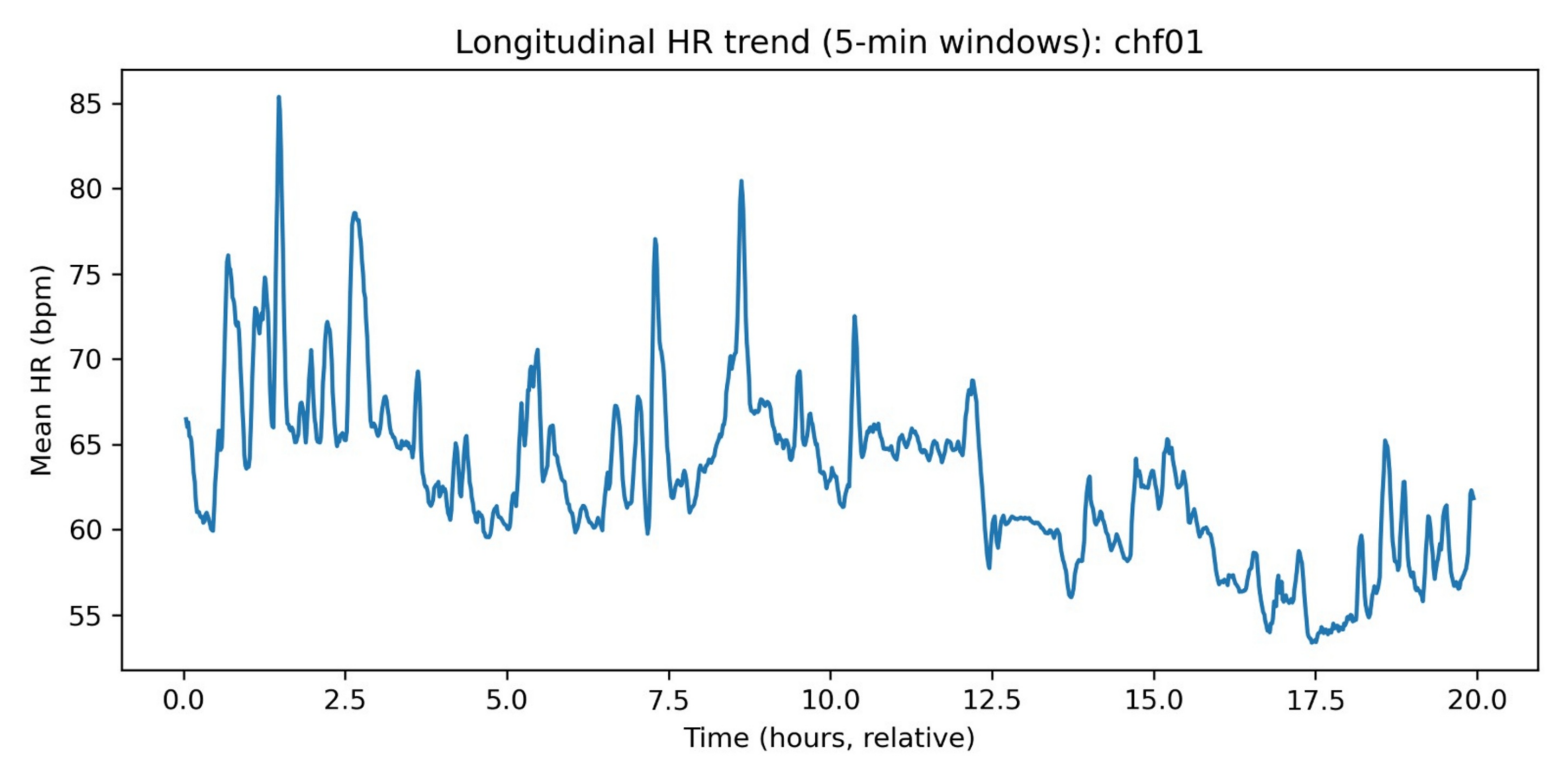
Longitudinal EDR trend (five-minute windows). EDR: ECG-derived respiration; HR: heart rate.

Figures 5, 6 present an illustrative example (chf01). The HR trace shows multi-hour drifts and shorter-term variability, while the EDR proxy shows a comparatively stable baseline with episodic excursions. EDR methods estimate respiratory dynamics from ECG via HR modulation, QRS amplitude variation, or morphology-based transformations; these approaches are widely studied and can be effective, but their accuracy is method-dependent and can degrade with motion artifacts or rhythm abnormalities [22,23].

In this paper, EDR is thus regarded as a respiratory dynamics measure as opposed to the gold-standard respiratory rate measure. These longitudinal plots are arguably the most clinically interpretable outputs of wearable-remote monitoring systems: the trend of multi-signal trajectories (cardiac rate/rhythm, respiratory pattern, activity capacity, congestion surrogates) is becoming the focus of current non-invasive monitoring approaches in heart failure to identify periods of decompensation days before overt deterioration [24-27]. Although the labelled endpoints (e.g., hospitalisation, mortality) were not given in this dataset, the presence of the inter-individual heterogeneity and within-subject temporal structure illustrates that one can extract the specific classes of the features targeted by the existing wearable or remote monitoring initiatives [24-27,30].

## Discussion

This descriptive research shows that cardiac and respiratory systems can be characterised in severe HF patients with long-duration ambulatory ECG, which is put in the perspective of the contemporary wearable and remote monitoring solutions. Based on the BIDMC CHF Database, our results demonstrate that the ECG-based HR, HRV, and EDR measures reveal large inter-individual heterogeneity and significant dynamics in longitudinal changes, even though there are no labelled clinical outcomes. Mean HR was spread across subjects and, in many cases, was associated with lower indices of HRV, such as SDNN and RMSSD. This observation aligns with the established evidence that advanced HF can be characterised by an autonomic imbalance, with increased sympathetic tone and weakened parasympathetic tone [3,4,13]. Notably, the non-significant correlation between mean HR and HRV in this cohort supports the fact that resting HR is an incomplete measure of autonomic state in HF in certain ambulatory settings where exercising, medication, and abnormal heart rhythms can impact HR levels [3,6]. The comparison was made between the early and late recording segments, showing that there was a general tendency for the mean HR of later parts of the recordings to be lower. This result is probably indicative of rest-activity rhythm and residual circadian modulation that are usually suppressed but not eliminated in HF, even though absolute circadian timing was not available [13]. According to a remote monitoring viewpoint, such findings underscore the importance of monitoring within-subject trends in comparison to cross-sectional thresholds only, a concept also gaining more and more force in the support of wearable-based HF management [9,10].

The frequency-domain HRV analysis revealed heterogeneous LF/HF distributions in the subjects. Using a different approach (appropriate for non-stationary and pathological scenarios) according to the current recommendations, LF/HF was viewed as a descriptive spectral characteristic but not necessarily an indicator of sympathovagal equilibrium. However, the fact that such measurements can be obtained using continuous ambulatory ECG underscores the richness that can be analysed through long-term measurements. The EDR analysis also revealed that the morphology of the ECG can be used to approximate the respiratory dynamics for long-term analysis. Longitudinal EDR patterns had constant baselines, but with sporadic variations with their previous approvals of respiratory values derived with ECG, plus their relevance in ambulatory tracking as low-burden respiratory proxies [7,8]. These findings, when coupled with HR trends, demonstrate how single-sensor systems could be used in multi-dimensional capture of physiological data applicable in the detection of HF. There are a number of constraints that must be mentioned. The sample is too small, and the clinical outcome data are not available to make any prognostic inferences. ECG ground was automated and not verified, so it may create noise in the metrics of RR. Also, historical ambulatory recording might not be an exhaustive representation of artefacts in modern consumer wearables. However, physiological principles and methods of analysis are also direct applications. Finally, this article shows the opportunity and applicability of continuous ECG-based monitoring to detect autonomic and cardiorespiratory activity in severe HF. The results add validity to the relevance of longitudinal, multi-parameter physiological profiling as a baseline for an upcoming wearable-based remote care plan. The relevance of remote physiological monitoring became particularly evident during the COVID-19 pandemic, which disproportionately affected patients with pre-existing HF while simultaneously disrupting healthcare delivery. Recent evidence highlights long-term cardiovascular sequelae of COVID-19 in this population, underscoring the need for validated noninvasive monitoring approaches during periods of system strain [31].

These findings should be interpreted in light of the study’s methodological and data limitations, reinforcing their role as descriptive insights rather than evidence of clinical effectiveness. First, the sample size was small (n = 15), limiting generalizability and precluding statistical inference regarding clinical outcomes. Second, the analysis relied on retrospective, publicly available data without access to detailed clinical variables such as medication dosing, comorbidities, or outcome events, which constrained interpretation to physiological characterization rather than prognostic modelling. Third, ECG annotations were automated and not manually verified, introducing potential error in RR interval detection and derived HRV measures. Fourth, the historical ambulatory ECG recordings may not fully capture motion artifacts and signal disturbances commonly encountered in contemporary consumer-grade wearable devices. Finally, the absence of labelled endpoints such as hospitalization or mortality prevented direct evaluation of predictive utility. These limitations should be considered when interpreting the findings and translating them to modern remote monitoring applications.

## Conclusions

This article shows that long-term ambulatory ECG monitoring is feasible and analytical in characterising cardiac and respiratory physiology in a patient with severe HF. We demonstrated with publicly available data at the BIDMC CHF Database that measures of HR, HRV, and respiration obtained by electrocardiography methods reveal a high level of inter-individual variability and significant within-subject time-dependent variations over significant periods of observation. There was less HRV and non-homogeneous autonomic profiles across the cohort, which are in line with higher advanced autonomic dysfunction in severe HF. Notably, longitudinal studies demonstrated dynamic aspects of changes in physiological parameters in individual recordings, which are the drawbacks of episodic measures and substantiate the importance of the continuum of monitoring to the study of disease physiology. The ability to extract respiratory proxies using the morphology of ECG is also an indication of how single-sensor systems can offer multi-dimensional physiology that can be used to support remote monitoring devices. Although this investigative analysis was not intended to measure clinical outcomes or predict indicative viability, the results outline the translational significance of ambulatory ECG as an underpinning of the new wearable and remote checks plans. Such practices may allow detecting physiological instability sooner and more individually treating HF by allowing continuous and noninvasive monitoring of cardiorespiratory measurements. Further work ought to involve how these physiological characteristics can be combined with contextual information, as well as confirmed clinical outcomes of their role and prognostic capability in informing prompt intervention.
